# EXIA2: Web Server of Accurate and Rapid Protein Catalytic Residue Prediction

**DOI:** 10.1155/2014/807839

**Published:** 2014-09-11

**Authors:** Chih-Hao Lu, Chin-Sheng Yu, Yu-Tung Chien, Shao-Wei Huang

**Affiliations:** ^1^Graduate Institute of Molecular Systems Biomedicine, China Medical University, Taichung 40402, Taiwan; ^2^Department of Information Engineering and Computer Science, Feng Chia University, Taichung 40724, Taiwan; ^3^Department of Medical Informatics, Tzu Chi University, Hualien 97004, Taiwan

## Abstract

We propose a method (EXIA2) of catalytic residue prediction based on protein structure without needing homology information. The method is based on the special side chain orientation of catalytic residues. We found that the side chain of catalytic residues usually points to the center of the catalytic site. The special orientation is usually observed in catalytic residues but not in noncatalytic residues, which usually have random side chain orientation. The method is shown to be the most accurate catalytic residue prediction method currently when combined with PSI-Blast sequence conservation. It performs better than other competing methods on several benchmark datasets that include over 1,200 enzyme structures. The areas under the ROC curve (AUC) on these benchmark datasets are in the range from 0.934 to 0.968.

## 1. Introduction

Enzymes play important roles in various biological processes. As the number of sequenced genomes rapidly grows, the function of the majority of proteins can only be annotated computationally. While a number of methods have been reported to predict protein function from protein sequence [1–3], protein structure [4, 5], protein-protein interaction network [6, 7], and evolutionary relationships [8–10], the complexity of protein function makes function prediction challenging. In addition, prediction of protein function is distinct from actual identification of functional regions or residues. To identify the location of functional regions in protein, a number of methods have been reported to predict protein functional site, including ligand binding sites [11–13], phosphorylation sites [14, 15], protein-protein interaction sites [16–18], and ubiquitination site [19] from protein sequence, structure, or high-throughput experimental data. Here we focus on the prediction of protein catalytic residues. Although only a small number of residues compose enzyme catalytic site, they are the most crucial part for enzymes to perform their function. Identifying these critical residues and characterizing their features are crucial to understanding the molecular basis of protein function.

Sequence or structure homology information is the primary feature used in catalytic residue prediction [20–26] because catalytic residues are usually evolutionarily conserved. One of the most successful sequence-based methods is CRpred [27], which used several types of sequence-based features including position-specific scoring matrix and entropy of weighted observed percentages extracted from multiple sequence alignment using PSI-BLAST [28]. Another method, ConSurf, identifies functionally important regions in proteins by estimating the degree of conservation of the amino acid sites among their close sequence homologues [29]. However, homology-based methods require reliable evolutionary information, for example, multiple sequence alignment constructed from enough number of protein sequences. Recent studies show that the evolutionary origin of one-third of genes is not clear in yeast genome [30]. For a protein that lacks reliable homology information, it is important to develop prediction method based on information contained in the protein itself. Several methods have been proposed to extract as much information as possible from protein structure. A method is based on the observation that catalytic residues are usually moderately exposed residues that are located closest to the protein centroid [31]. It was shown that if a protein was converted to a network in which the residues are vertices and their interactions are edges, the central hubs are usually functionally important residues or their neighboring residues [32]. It was also reported that catalytic residues usually have higher force constant, that is, the ease of moving a given residue with respect to the other residues in the protein [33]. The theoretical microscopic titration curves (THEMATICS) [34] method detects catalytic residues by calculating theoretical residue electrostatic properties from protein structure. THEMATICS was then enhanced by structure geometric features [35] to detect catalytic residues from protein structure using a monotonicity-constrained maximum likelihood approach, called partial order optimum likelihood (POOL). A more recent study [36] models the properties, such as physicochemical properties, atomic density, flexibility, and presence of water molecules or heteroatom, of spherical regions around target residues. Such methods are helpful for proteins that do not have reliable homology information. However, current methods that do not rely on homology information perform worse than other homology-based methods.

Here we propose a method (EXIA2) of catalytic residue prediction based on protein structure without needing homology information. The method is an improved version of our previous work [37], which is based on the special side chain orientation of catalytic residues. We found that the side chain of catalytic residues usually points to the center of the catalytic site. The special orientation is usually observed in catalytic residues but not in noncatalytic residues, which usually have random side chain orientation. The feature is effective in the identification of catalytic residues from enzyme structure. In this work, we further add a new property, the amino acid combination feature, which is a general composition of amino acids in enzyme catalytic site. We implement the web server and optimize its computation efficiency for practical use. The prediction performance of EXIA2 web server is better than those of other state-of-the-art prediction methods. In addition to better prediction performance, it is more efficient than other structure-based prediction methods.

## 2. Description of Web Server

### 2.1. Input

The input for the web server is a 3D protein structure in Protein Data Bank (PDB) [38] format. Users can upload their own protein structure file or input a PDB id. Each submission allows a structure of up to 5000 residues. The results are displayed instantly for small and medium size proteins. For proteins of larger size (more than 3000 residues), the processing time is normally from several seconds to a minute.

### 2.2. Output

**Figure 1:**
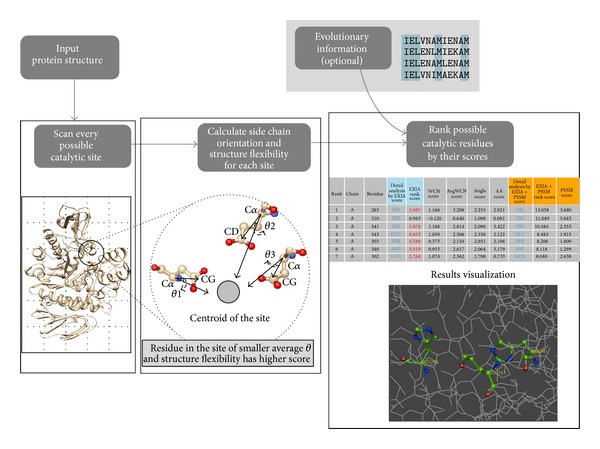
Overview of the EXIA2 web server. The input of the EXIA2 web server is a protein structure. EXIA2 first scans all possible catalytic sites in the structure and then computes the side chain orientation of residues in each candidate site. A residue receives higher score when it and its neighboring residues have their side chains pointing to their center position. The structure flexibility of residues is also included in scoring. After the structure-based calculation is finished, users may optionally add the sequence conservation from PSI-Blast, which usually takes longer calculation time. The final results are the possible catalytic residues ranked by their scores and all the detailed scores (side chain orientation score, structure flexibility score, and sequence conservation score) calculated in the prediction process.

The web server first predicts possible catalytic residues based on information extracted from the input structure. Users can optionally choose to combine the structure-based results with evolutionary information from PSI-Blast position-specific substitution matrix. The web server provides a one-click link for users to submit a PSI-Blast search and the evolutionary information is automatically combined with the structure-based prediction results when PSI-Blast search is finished. The computation results include possible catalytic residues ranked by their scores, which are calculated based on various sequence and structure features. The detailed scoring of each feature is also provided for users to judge and interpret the prediction results. In addition to raw computation data, the web server visualizes structures around the predicted catalytic residues for users to easily inspect the regions in which they are interested.  Figure 1 shows a brief overview of the web server.

## 3. Methods

The method uses the following features to predict catalytic residues: residue side chain orientation, theoretical structural flexibility, and amino acid combination. The success of the method is to scan one small region of the structure at a time, instead of just considering the properties of each single residue. For each region that is probable to be catalytic site, we calculate the side chain orientation of residues in the region. A region is more probable to be catalytic site if the side chain of residues in the region tends to point to the center of the region. In addition to side chain orientation, we also calculate the structure flexibility and amino acid combination for each region. The following sections explain the detailed calculations.

### 3.1. Side Chain Orientation

A vector *s*_*k*_ is defined as the side chain direction of residue *k*:

(1) $$\begin{array}{c} s_{k}={Χ}_{k}^{F}-{Χ}_{k}^{\text{CA}}, \end{array}$$

where X_*k*_^*F*^ and X_*k*_^CA^ are the crystallographic position of the side chain vector atom and C*α* atom of residue *k*. The side chain vector atom is carefully chosen for each amino acid type. It is either the most frequent functional atom defined in catalytic site atlas (CSA) [39] or the atom located near the centroid of multiple possible functional atoms. Here, side chain vector atoms are only defined for residues whose functional atom is usually on its side chain: Arg: CZ, Asn: CG, Asp: CG, Cys: SG, Gln: CD, Glu: CD, His: NE, Lys: NZ, Ser: OG, Thr: OG, Trp: CZ, and Tyr: OH. Only amino acid types defined here are included in the calculations of side chain orientations.

### 3.2. Prediction Procedure

All nonprotein ligands are removed from the input structure. The structure is then embedded in a three-dimensional 30 × 30 × 30 grid of points. The reason of using fixed grid spacing is that we want to make sure the server finishes the calculations in reasonable time even for larger proteins. The grid size is the optimal balance between computation time and prediction performance. The grid spacing is from 1.33 angstrom to 2.13 angstrom depending on the protein size and is small enough to scan possible catalytic site even for large proteins. Each grid point is a probable position of catalytic site. For each grid point *i*, the* surrounding residues* of point *i* are the residues whose distance between its C*α* atom and point *i* is less than 10Å. Grid points with less than three surrounding residues are removed. For each point *i* and any one of its surrounding residues *j*, the vector between point *i* and C*α* atom of residue *j* is defined as

(2) $$\begin{array}{c} v_{ij}={Χ}_{i}-{Χ}_{j}, \end{array}$$

where *Χ*_*i*_ and *Χ*_*j*_ are the position of point *i* and C*α* atom of residue *j*. We compute the angle *θ*_*ij*_ between *v*_*ij*_ and *s*_*j*_, which is the side chain vector of residue *j*:

(3) $$\begin{array}{c} \theta_{ij}=\;\text{acos}\;\frac{v_{ij}\cdot s_{j}}{||v_{ij}||||s_{j}||}. \end{array}$$

For a grid point within the area of the catalytic site, its surrounding residues usually have smaller *θ* angles. Based on this observation, we calculate the averaged angle *θ*_*i*_ among all of the surrounding residues for point *i*, as in

(4) $$\begin{array}{c} \theta_{i}=\sum \frac{\theta_{ij}}{N}, \end{array}$$

where *N* is the number of surrounding residues of point *i*. We assume that points near catalytic site have smaller averaged *θ* and remove the points that have averaged *θ* > 80°. The cut-off value is chosen based on a statistics of *θ* angles for all residues in the PW79 dataset (as shown in Figure S1 in Supplementary Material available online at http://dx.doi.org/10.1155/2014/807839). About 80% of catalytic residues have the angle *θ* ≤ 80 degrees. We tried different cut-off values ranging from 30 to 100 degrees and found that the prediction performance is the best when the cut-off value is 80 degrees on the PW79 dataset. For every remaining point (points with *θ* ≤ 80°), we select three residues at a time from its surrounding residues and give each selected residue a score (referred to as* feature score*) according to their features. For each point, the selection process is repeated for all possible combinations of any three surrounding residues (referred to as triplet). Each time a residue is involved in a selected triplet, the residue receives a feature score based on the features of selected triplets. A residue is possible to be the surrounding residue of multiple grid points and therefore involved in triplets that belong to different grid points. An example of score calculation is available in supplementary Figure S2. Residues are finally ranked by their sum of all feature scores (denoted by *S*) received from all grid point that includes the residue. The final result is a list of residues ranked by their *S* score, that is, the likelihood of being a catalytic residue according to our prediction.

### 3.3. Feature Scores

The feature score is calculated based on theoretical structural flexibility and amino acid combination of residues. The weighted-contact number model (WCN) [41, 42] is used to compute structural flexibility. Catalytic residues usually have more rigid structures, that is, having higher WCN [43–45]. B-factor is not directly used because, in many cases, crystal structures of the same enzyme have almost identical 3D structure but have quite different B-factor profiles. In addition, B-factor is only available for structures solved by X-ray crystallography but not available for structures solved by NMR. WCN is more reliable than B-factor to measure the structural flexibility. For a residue *k* in a structure, its WCN *w*_*k*_ is defined as

(5) $$\begin{array}{c} w_{k}=\sum_{m\neq k}\frac{1}{r_{km}^{2}}, \end{array}$$

where *m* is any other residues in the structure and *r*_*km*_ is the distance between the C*α* atoms of residues *k* and *m*. The WCN scores are normalized as in

(6) $$\begin{array}{c} z_{k}^{w}=\frac{w_{k}-\overset{-}{w}}{\sigma }, \end{array}$$

where *z*_*k*_^*w*^ is the normalized WCN of residue *k* and $\overset{-}{w}$ and *σ* are the mean and standard deviation of the WCN of all residues in the protein. As described in the previous section, for every remaining point with *N* surrounding residues, we select three residues (triplet) from surrounding residues and give each selected residue a feature score. The purpose is to give higher score to residues involved in “better” combination, that is, triplet that are more structurally rigid. For a selected triplet (denoted by *n*, a subset of the *N* surrounding residues), we define an averaged WCN *w*_*n*_, which is the average structure flexibility of these three residues:

(7) $$\begin{array}{c} w_{n}=\sum_{j\in n}\frac{z_{j}^{w}}{3}, \end{array}$$

where *z*_*j*_^*w*^ is the normalized WCN, *w*_*j*_, of residue *j*. Among the three residues, each residue receives a feature score *S*:

(8) $$\begin{array}{c} S=w_{n}+z_{j}^{w}+z_{j}^{a}, \end{array}$$

where *w*_*n*_ is the averaged WCN, *z*_*j*_^*w*^ is the normalized WCN of residue *j*, and *z*_*j*_^*a*^ is the normalized amino acid combination score of residue *j*. The amino acid combination score is based on statistics of the amino acids of catalytic sites in the PW79 dataset [37]. For each type of amino acid, a profile *p*_*x*_ containing 20 elements is constructed:

(9) $$\begin{array}{c} p_{x}=\left(p_{x}^{\text{ALA}},p_{x}^{\text{CYS}},p_{x}^{\text{GLY}},\ldots ,p_{x}^{\text{VAL}}\right), \end{array}$$

where *p*_*x*_ denotes the profile of amino acid type *x*; each element in the profile is the frequency of an amino acid type appearing in the same catalytic site as amino acid type *x*. Here residues are defined as in the same catalytic site if they are all annotated as catalytic residue and located in the same catalytic site defined by the CSA database. The *z*_*j*_^*a*^ score in (8) is calculated as in

(10) $$\begin{array}{c} z_{j}^{a}=\frac{a_{j}-\overset{-}{a}}{\sigma^{a}}=\frac{\left(p_{x}^{y}+p_{x}^{z}\right)-\overset{-}{a}}{\sigma^{a}}, \end{array}$$

where *a*_*j*_ denotes the amino acid combination score of residue *j* and $\overset{-}{a}$ and *σ* ^*a*^ are the mean and standard deviation of the amino acid combination score of all residues in the protein. *a*_*j*_ is the sum of *p*_*x*_^*y*^ and *p*_*x*_^*z*^, where *x* is the amino acid type of residue *j* and  *y* and *z* are the amino acid types of the other two residues in the subset *n*, which has three selected residues as described previously.

Most catalytic residues have their functional atom on the side chain; there are about 5% of catalytic residues that have functional atom on the backbone. These catalytic residues are usually hydrophobic and nonpolar amino acids and are not involved in the calculations of side chain orientations. In the results of the web server, we provide users the structural flexibility and amino acid combination scores for these residues.

### 3.4. Evolutionary Information

The method becomes more powerful by including evolutionary information. Users may include evolutionary sequence conservation from PSI-Blast [28] position-specific substitution matrix (PSSM) to the prediction. EXIA2 web server provides users a one-click option to submit PSI-Blast search on the web server. The sequence conservation information is automatically combined with structure-based features. PSI-Blast is set to search against the nonredundant (nr) database for three iterations with an *E*-value threshold of 5 × 10^−3^. The nr database is a default built-in protein sequence database in PSI-Blast. The sequence conservation score *c*_*j*_ of residue *j* is directly taken from the “information per position” column in PSSM. The sequence conservation score *c*_*j*_ is included in the final score *S*_*j*_ of residue *j* as in

(11) $$\begin{array}{c} S_{j}^{\prime }=S_{j}+1.6\times z_{j}^{c}, \end{array}$$

where *S*_*j*_ is the final score of residue *j* based on structure information and *z*_*j*_^*c*^ is the normalized *c*_*j*_ of residue *j* as in

(12) $$\begin{array}{c} z_{j}^{c}=\frac{c_{j}-\overset{-}{c}}{\sigma^{c}}, \end{array}$$

where *c*_*j*_ is the original sequence conservation score of residue *j* and  $\overset{-}{c}$ and *σ* ^*c*^ are the mean and standard deviation of all the sequence conservation scores in the protein. The parameter in (11) was tuned based on the PW79 dataset. The prediction performance (AUCROC) is optimal for the dataset when the value is set to 1.6.

### 3.5. Datasets

The PW79, POOL160, EF, and P100 datasets are from [35, 40, 46, 47], respectively. The proteins in the L55 dataset (Table S1) are selected from the PW79, POOL160, and EF datasets. Among all proteins in these datasets, we first picked the proteins without bounding ligand (78 proteins selected). Then for each protein, a structure that has the most similar sequence (most of them have completely the same sequence) and has bounding ligand in the catalytic pocket was selected from the PDB database. 23 proteins among the 78 structures that have no available structure with bounding ligand in PDB are removed. There are totally 55 pairs of enzyme structures selected as the L55 dataset. The EX79 dataset (Table S2) is built by combining all proteins in the POOL160 and EF datasets and excluding all proteins that are in the PW79 dataset. The definition of catalytic residues is based on Catalytic Site Atlas version 2.2.12.

## 4. Performance

We compared the prediction performance of EXIA2 web server with that of three state-of-the-art prediction methods on six benchmark datasets [37], PW79, POOL160, EF fold, EF superfamily, EF family, and P100 which include over 1,200 proteins and 861,404 residues (3,664 catalytic residues and 857,740 noncatalytic residues). Tables 1 and 2 summarize the comparison of prediction performances, including recall (R), precision (P), and area under ROC curve (AUCROC).True positives are correctly predicted catalytic residues; false positives are noncatalytic residues incorrectly predicted to be catalytic residues; true negatives are correctly predicted noncatalytic residues; false negatives are catalytic residues incorrectly predicted to be noncatalytic residues. For each protein, we calculate the prediction result under different cut-off values (true positive rate from 0 to 1) and draw the ROC curve and recall-precision curve. The overall prediction result of a dataset is by averaging the per-protein ROC curves (or recall-precision curves) in the dataset. When comparing the prediction performance with other methods, the recall and precision values are directly retrieved from the overall recall-precision curve for a dataset.

### 4.1. Comparison with Other Methods

Table 1 compares the prediction results of EXIA2 and the results of a prediction method [36], which uses many sequence, structure, and evolutionary features to model residue structural neighborhood. The prediction performance of EXIA2 combined with PSI-Blast evolutionary information (PSSM) is better than that of the competing method. Among the six benchmark datasets, the recall (or precision) is higher than that of the competing method when the precision (or recall) is equal to theirs. EXIA2 also has higher AUCROC than theirs in the PW79 and POOL160 datasets (the AUCROC for the other three datasets are not provided in the report of the competing method).

**Figure 2:**
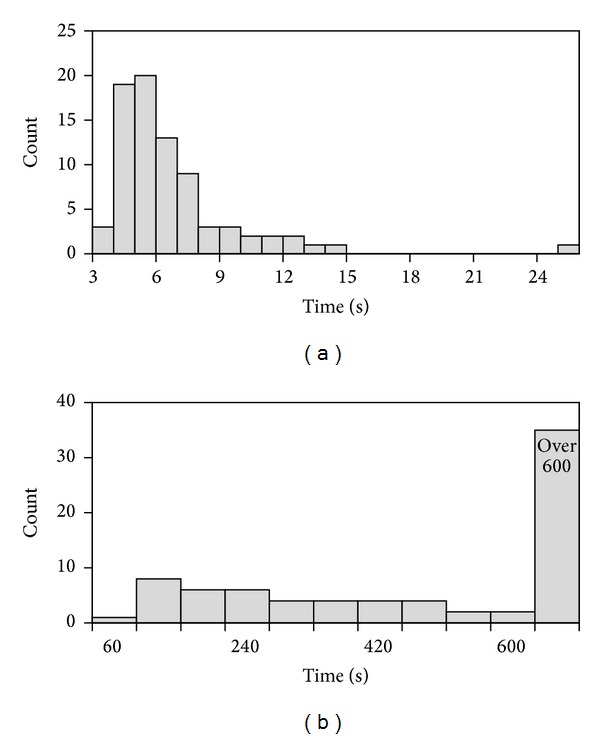
Distribution of computation time for the PW79 dataset. The figure shows the computation time for (a) EXIA2 web server and (b) POOL web server.

We also compare the prediction results of EXIA2 web server* without* using PSSM information and those of two other prediction methods: POOL that uses only structure information and CRpred [27] that uses only sequence information. We compared the precision when our recall is equal to theirs and the recall when our precision is equal to theirs (Table 2). The results show that EXIA2 performs better than these two methods. It has higher recall (70.8) and higher precision (20.2) when the precision and recall a0re equal to those of POOL (18.1 and 61.7, resp.) for the POOL160 dataset. Most current prediction methods do not perform well when only structure-based features are used. Evolutionary information is usually required for prediction methods to have better prediction results. POOL [35], which calculates theoretical residue electrostatic property and structure shape, is one of the best structure-based prediction methods. EXIA2 performs better than POOL for the POOL160 dataset. The results indicate that EXIA2, which uses side chain orientation and structure flexibility, is more effective than the structure features used by POOL. In addition to prediction performance, EXIA2 web server is more computationally efficient than POOL web server [48]. The prediction results of EXIA2 web server are usually displayed instantly. POOL web server usually needs several minutes to finish the calculations. We compared the computation time used by EXIA2 and POOL by submitting the 79 proteins in the PW79 dataset to the two web servers. For the POOL web server, the submission of three proteins (PDB ID: 1B57, 1DCO, and 1DQS) did not finish correctly (server crash due to parameter errors during calculation). These proteins are excluded in the comparison. The average computation time of EXIA2 is 6.25 seconds and that of POOL server is 868.14 seconds (the time for generating PSI-Blast profile not included). For POOL web server, the computation time of 46% of proteins in the dataset is more than 600 seconds. For EXIA2 web server, the maximum computation time is 25 seconds on a protein of about 3000 residues. The results show that EXIA2 web server is very efficient and stable.  Figure 2 shows the distribution of computation time for the EXIA2 and POOL web server for the PW79 dataset.

CRpred is currently the best sequence-based catalytic residue prediction method. In the prediction of protein catalytic residue, prediction results using only sequence information are usually much better than the results only using structure information. The reason may be that sequence information includes evolutionary conservation and catalytic residues are usually highly evolutionarily conserved. Here, the prediction results of EXIA2 without adding evolutionary conservation are better than those of CRpred. EXIA2 has higher recall (67.8) and higher precision (24.7) when the precision and recall is equal to those of CRpred (17.5 and 53.7, resp.) for the PW79 dataset. It also performs better than CRpred on the EF fold dataset. Although EXIA2 has slightly smaller recall and precision values than those reported by CRpred (Table 2), it still performs better than CRpred by looking at their ROC curve (Figure S3), which is a much more complete performance measure. The results show that the structure features used by EXIA2 are very effective.

We also compare the performance of EXIA2 with that of ConSurf [29], ResBoost [40], and a recent structure-based prediction method [49] on two test datasets. ConSurf and ResBoost are both based on evolutionary conservation, various sequence, and structure features. ConSurf identifies functionally important regions in proteins by estimating the degree of conservation of the amino acid sites among their close sequence homologues. ResBoost predicts catalytic residues based on several features, including evolutionary conservation, 3D clustering, residue solvent accessibility, and hydrophobicity. EXIA2 server performs better than both ConSurf and ResBoost even without using sequence conservation information (Table 2) on the P100 dataset. The comparisons of their ROC curves and recall-precision curves are available in supplementary Figure S4. Another recent structure-based prediction method [49] is based on various centrality measures of nodes in graphs of interacting residues: closeness, betweenness and page-rank centrality, general center of mass of the structure, relative solvent accessibility, and sequence conservation. EXIA2 also performs better than the method on a test set of 29 proteins. EXIA2 has higher precision (21.7 versus 17.1) when the sensitivity is equal to theirs and has higher sensitivity (71.9 versus 63.1) when the precision is equal to theirs. However, the prediction results of the method without sequence conservation are not available on the test dataset.

### 4.2. Performance for Enzyme Structure without Bounded Ligand

**Figure 3:**
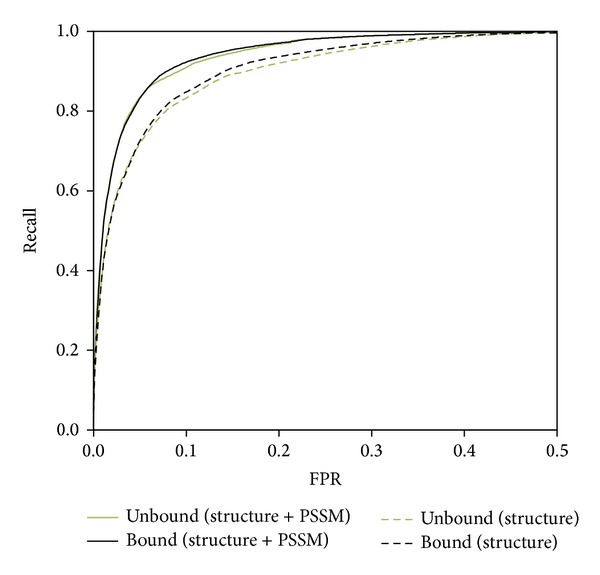
ROC curves for L55-Bound and L55-Unbound enzyme structures. Comparison of performance on 55 enzymes in their substrate-bounded (L55-Bound) form and substrate-unbounded form (L55-Unbound). The performances of L55-Unbound are similar to those of L55-Bound using only structure features (dashed lines) or using both structure features and sequence conservation (solid lines). The AUCROC for L55-Bound and L55-Unbound are 0.968 and 0.967, respectively, when both structure features and sequence conservation are used. The AUCROC are 0.950 and 0.947 for L55-Bound and L55-Unbound, respectively, when only structure features are used.

**Figure 4:**
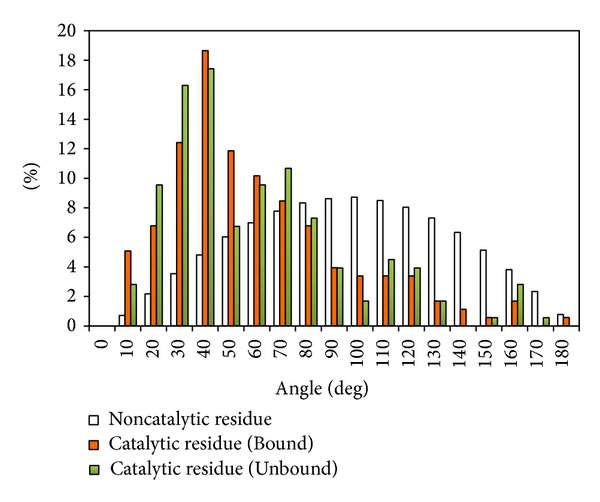
Comparison of side chain orientation of catalytic and noncatalytic residues. Comparison of side chain orientation for catalytic residues in structures of L55-Bound (orange bar) and L55-Unbound (green bar) set and random selected noncatalytic sites (white bar). Residues with small angles have their side chain pointing to the center of the site (see Figure 1 for the definition of angle calculation).

We construct a dataset (L55, PDB IDs listed in Table S1) that contains 55 enzymes and their structures crystallized with substrates (denoted by L55-Bound) and without substrates (L55-Unbound).  Figure 3 shows the ROC curves for the structures of L55-Bound and L55-Unbound. The performances of EXIA2 on these two sets of structures are very similar. The AUCROC for L55-Bound and L55-Unbound are 0.968 and 0.967, respectively, when both structure features (side chain orientation and flexibility) and sequence conservation are used. The AUCROC are 0.950 and 0.947 for L55-Bound and L55-Unbound, respectively, when only structure features are used to perform prediction. The results suggest that the special side chain orientation of catalytic residue exists not only in substrate-bounded structures but also in structures without bounded ligands. In Figure 4, we further analyze the angle between the side chain vector of catalytic residue and the vector of the residue C*α* atom to the center of the catalytic site (as the *θ* angle described in Figure 1 or (3)). Residues whose side chain tends to point to the center of the catalytic site have smaller angles. For catalytic residues of both substrate-bounded (orange bar) and substrate-unbounded (green bar) structure, their angles are smaller than those of noncatalytic residues. For the angle calculation of noncatalytic residues, we randomly pick noncatalytic residues and include its structurally neighboring residues within 10 angstroms. For each random “noncatalytic site” selected, we calculate the angle between the side chain vector of these residues and the vector from their C*α* atom to the center of the site. The results indicate that the special side chain orientation only exists in catalytic residues but not in noncatalytic residues. More importantly, the results also suggest that the side chain structures of catalytic residues are ready to interact with substrates even before substrate binding. The observation also explains the success of EXIA2 to identify the catalytic residues for enzymes without bounded ligands.

### 4.3. Effect of Amino Acid Combination Feature

**Figure 5:**
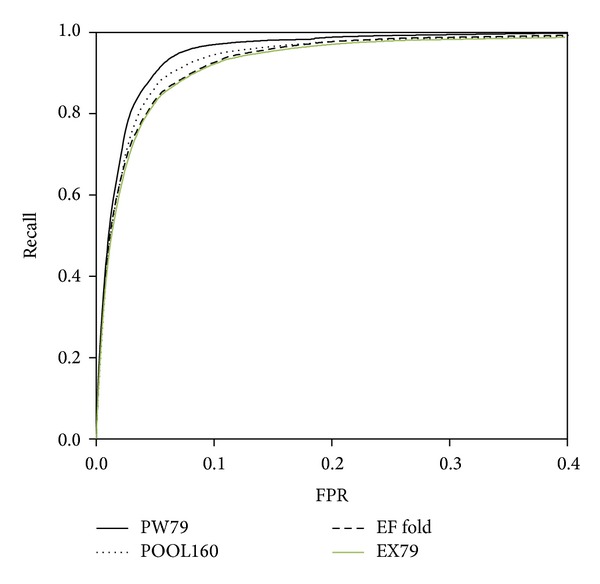
ROC curves for PW79, POOL160, EF fold, and EX79 dataset. The prediction performance using structure features combined with sequence conservation. The AUCROC for the PW79, POOL160, EF fold, and EX79 dataset are 0.973, 0.965, 0.968, and 0.964, respectively.

In the EXIA2 server, we add the amino acid combination feature, which is a general composition of amino acid types in enzyme catalytic sites. The scoring of the feature is calculated based on the enzymes in the PW79 dataset. To evaluate the performance of the feature, we construct a dataset (EX79 dataset, PDB IDs listed in Table S2) that combines the POOL160, EF fold, EF superfamily, and EF family datasets and excludes all of the enzymes from the PW79 dataset.  Figure 5 shows the receiver operating characteristic (ROC) curve for the PW79, POOL160, EF fold, and the EX79 dataset. The ROC curve of EF family and EF superfamily is similar to that of EF fold and not shown in the figure. The results show that the performance (AUCROC = 0.964) of the EX79 dataset is similar to that of the EF fold dataset (AUCROC = 0.968). It suggests that the feature is still effective for enzymes that were not used to calculate the amino acid combination feature.

To see the effect of amino acid combination feature on the prediction performance, we compare the AUCROC of prediction using structure feature only and using both structure feature and amino acid combination. The AUCROC is improved from 0.938 to 0.944 on the EX79 dataset. Figure S5 shows the ROC curve of prediction with and without amino acid combination feature on the EX79 dataset. The TPR values are improved especially when FPR is smaller than 0.15. EXIA2 is primarily based on the intrinsic structure features, side chain orientation, and structure flexibility, of the input protein. In this work, the amino acid combination feature is added to the web server because of its practical usage to identify possible catalytic site.

## 5. Prediction Examples

### 5.1. Human Ferrochelatase

**Figure 6:**
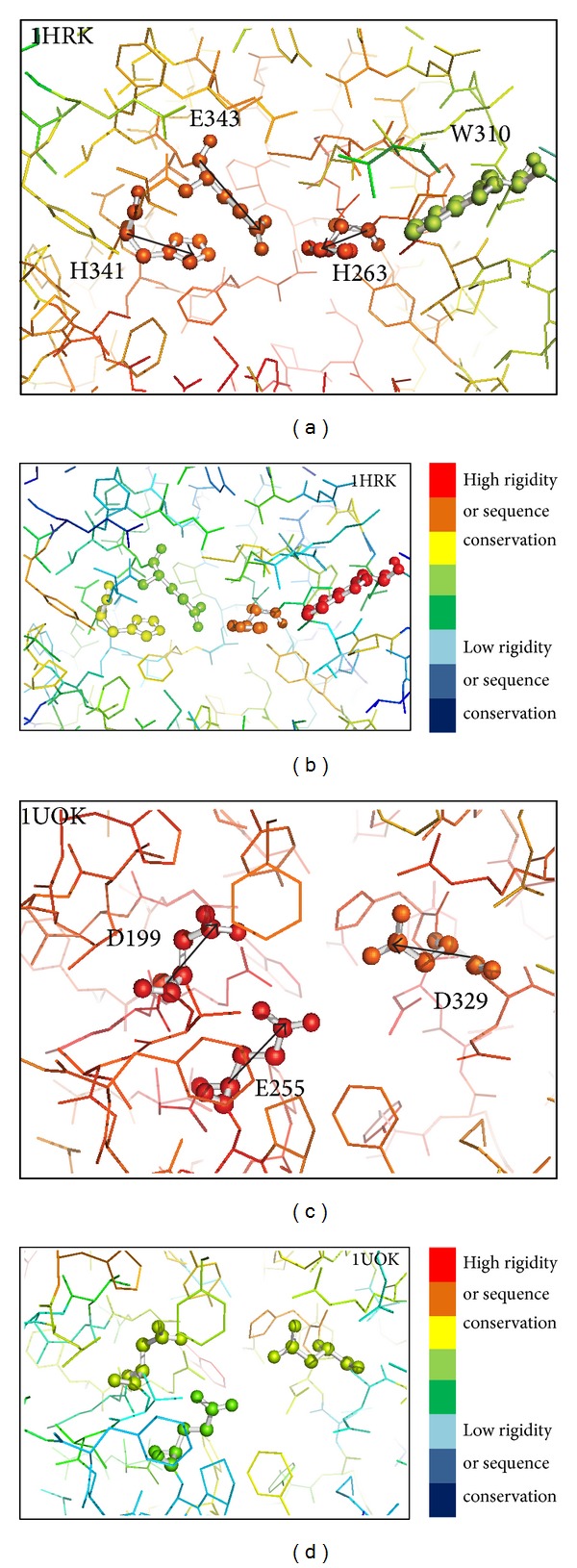
Structures around the catalytic residues of two example enzymes colored by residue structure flexibility and sequence conservation. The structures around the catalytic residues of Human ferrochelatase (PDB: 1HRK) and Oligo-1,6-glucosidase (PDB: 1UOK). The rainbow coloring in (a) and (c) is from blue (low structure rigidity or high structure flexibility) to red (high structure rigidity or low structure flexibility). (b) and (d) are colored from blue (low sequence conservation) to red (high sequence conservation). Structure flexibility and sequence conservation are based on the WCN model and PSI-Blast PSSM as described in Section 3. The black arrows in the figure indicate the direction of side chain vector for the catalytic residues.

**Figure 7:**
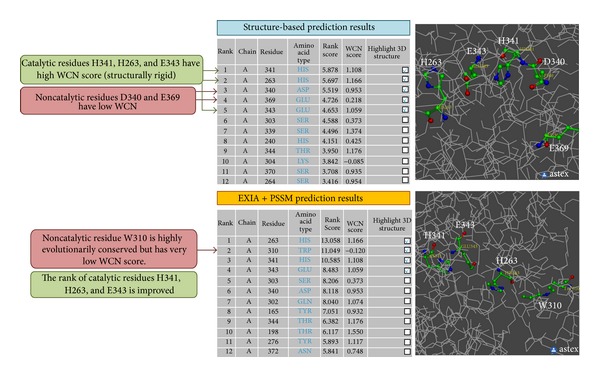
Server output results of protein Human ferrochelatase. The figure shows part of the output results of the EXIA2 web server for Human ferrochelatase (PDB: 1HRK). The main results are possible catalytic residues predicted by the server ranked by their rank score. The prediction results are improved when evolutionary information from PSI-Blast PSSM is included in the prediction. The WCN score of each residue is also provided for users to further analyze the results. The detailed scores, including side chain orientation score, average structure flexibility, and PSSM scores, used in the prediction are also provided (not shown here).

The catalytic site of Human ferrochelatase (PDB ID: 1HRK) includes three catalytic residues, H263, H341, and E343. Figures 6(a) and 6(b) show the structures of the catalytic site and demonstrate a good example of the side chain orientations of catalytic residues. The side chain of the three catalytic residues point to the center of the catalytic site to interact with the ligand (ligand information is not used in the prediction). Catalytic residues H341, H263, and E343 are ranked 1st, 2nd, and 5th, respectively, in the prediction using only structure information. The output results are shown in Figure 7. Although the two noncatalytic residues, D340 and E369, are ranked 3rd and 4th, they have low WCN score (more flexible structure) and are less likely to be catalytic residues. The prediction results are further improved by adding evolutionary information (PSI-Blast PSSM). Catalytic residues H263, H341, and E343 are ranked 1st, 3rd, and 4th, respectively (Figure 7). The noncatalytic residue W310 is ranked 2nd because it is extremely evolutionarily conserved (Figure 6(b)). However, it has very low WCN score and is less probable to be catalytic residue (Figure 6(a)). The catalytic residues of the enzyme are correctly predicted because they have stable structure and proper side chain orientations. One of the successful designs of EXIA2 is that we consider not only the properties of single residue but also the properties of its neighboring residues. A residue receives high score when the residue and its neighbors have their side chain pointing to their centroid position and their average structure flexibility is low.

### 5.2. Oligo-1,6-glucosidase

The catalytic residues of oligo-1,6-glucosidase (PDB ID: 1UOK) are D199, E255, and D329. They are the top three ranked residues in the prediction results using only structure information. Each of the three catalytic residues has low structural flexibility (Figure 6(c)). In addition, they also have high average WCN score, which means that these residues and their neighboring residues form very stable structures. The enzyme shows a good example on the effect of calculating* average WCN score*. There are several noncatalytic residues that have better WCN score (the structure flexibility of the residue itself) than the three catalytic residues, but these catalytic residues have higher average WCN score (the average structure flexibility of the residue and its neighboring residues) than all the other residues in the enzyme. It suggests that considering the structural flexibility of single residue is not enough in the prediction of catalytic residues. The three catalytic residues also have extremely high side chain orientation score; that is, these residues and their neighboring residues have side chains pointing to their centroid (Figure 6(c)). The side chain orientation score of these catalytic residues is higher than those of all the other residues in the enzyme. Side chain orientation score helps to easily identify the most probable catalytic residues in this example. It also suggests that the side chain orientation feature is unique enough to be used in the prediction of catalytic residues because noncatalytic residues do not seem to have such property.

## 6. Conclusion

EXIA2 is an accurate and efficient catalytic residue prediction method. In addition to accurate identification of catalytic residues, the web server provides detailed scoring data, including the side chain orientation, structural flexibility, amino acid combination, and sequence conservation scores, for users to inspect and analyze the enzyme structure.The advantage of EXIA2 is that it does not rely on sequence or structure homology information. The fundamental feature used in EXIA2 is to detect the regions in which the residues' side chain points to the center of the region. We found that the special side chain orientation is usually observed for catalytic residues but not for noncatalytic residues. The prediction performance based on the phenomenon is better than those of existing prediction methods and is tested on various datasets, including a dataset of enzymes that do not have any bounded ligand in their crystallographic structures. The results suggest that the special side chain orientation exists not only in ligand-bounded structure but also in the apo form of enzymes.

EXIA2 is different from other competing machine learning methods (except POOL, which is also a heuristic-based approach). The performance of EXIA2 is mostly contributed from the intrinsic properties of input structure, the side chain orientation, and structure flexibility feature. There is no training process required to calculate these structure features. The prediction performance only based on these structure features is more accurate than those of other existing structure-based methods. Although there are few parameters that need to be optimized, most of them are based on statistics and observation of general enzyme properties. We also used the EX79 dataset, which exclude the 79 proteins used for parameter optimization, to test the performance of EXIA2. The results show that performance on EX79 dataset is similar to those of the EF fold, EF family, and EF superfamily datasets.

## Supplementary Material

Figure S1 Side chain orientation for catalytic and noncatalytic residues of PW79 dataset: Distribution of the angle between residue side chain vector and the vector of residue C alpha atom to the center point of catalytic site (center point of selected residues for noncatalytic residue).
Figure S2 Examples of triplet selection and score calculations: Detailed score calculation and triplet selection of an example structure including nine grid points.
Figure S3 Comparison of ROC curves of EXIA2 and CRpred: Comparison of ROC curves of EXIA and CRpred on the EF fold dataset. EXIA, with or without including PSSM, performs better than CRpred.
Figure S4 Comparison of ROC and RP curves of EXIA2, ResBoost, and ConSurf: Comparison of ROC and RP curves of EXIA, ResBoost, and ConSurf on the P100 dataset. EXIA, with or without including PSSM, performs better than the other two methods.
Figure S5 ROC curve of prediction with and without amino acid combination feature on the EX79 dataset: To see the effect of amino acid combination feature on the prediction performance, we compare the AUCROC of prediction using structure feature only and using both structure feature and amino acid combination. The AUCROC is improved from 0.938 to 0.944.
Table S1 List of PDB IDs and chain for 55 pairs of structures of the L55 dataset: 55 pairs of enzyme structures selected from the PW79, POOL160, and EF datasets. Each pair contains a structure with bounding ligand and a structure without bounding ligand.
Table S2 List of PDB IDs and chain of EX79 dataset: To test the prediction performance on a dataset excluding all structures used for parameter optimization (PW79 dataset), we construct the EX79 dataset by combining all proteins in the POOL160, and EF datasets and excluding all proteins of the PW79 dataset.

## Figures and Tables

**Table 1:** Comparison of prediction performance of EXIA2 web server and competing method using both sequence and structure information.

	Benchmark datasets				
	PW79	POOL160	EF fold	EF superfamily	EF family
Competing method^1^					
Recall (R)	46.0	78.1	64.2	67.3	61.7
Precision (P)	28.0	19.0	17.1	16.9	18.5
AUCROC	0.963	0.948	—	—	—
EXIA2 + PSSM^2^					
Recall at equal P	63.3	77.8	71.0	70.4	64.3
Precision at equal R	36.5	18.7	19.4	17.7	19.6
AUCROC	0.973	0.965	0.968	0.968	0.968
EXIA2^3^					
Recall at equal P	48.8	68.6	43.8	46.9	42.2
Precision at equal R	30.5	14.5	12.0	11.6	12.9
AUCROC	0.962	0.960	0.943	0.944	0.946

^1^Prediction results of Cilia and Passerini [36].

^2^Prediction results of EXIA2 combined with PSI-Blast PSSM.

^3^Prediction results of EXIA2 without evolutionary information.

**Table 2:** Comparison of prediction performance of EXIA2 web server and CRpred, POOL, ConSurf, and ResBoost.

Competing method	CRpred	POOL^1^	POOL^2^	ConSurf^3^	ResBoost^3^
Benchmark datasets	EF fold	POOL160	POOL160	P100	P100
Recall (R)	48.2	61.7	64.7	55.0	55.0
Precision (P)	17.0	18.1	19.1	5.0	17.0
AUCROC^4^	—	0.907	0.925	—	—
EXIA2 + PSSM^5^					
Recall at equal P	72.7	80.0	77.8	96.0	74.3
Precision at equal R	27.3	24.3	23.3	25.3	25.3
AUCROC	0.968	0.965	0.965	0.966	0.966
EXIA2^6^					
Recall at equal P	45.1	70.8	68.6	90.6	58.0
Precision at equal R	16.2	22.2	20.8	18.3	18.3
AUCROC	0.943	0.960	0.960	0.952	0.952

^1^Prediction results of POOL.

^2^Prediction results of POOL combined with evolutionary information.

^3^Prediction results published in [40]. Complete comparison of ROC and recall-precision curves is available in supplementary Figure S4.

^4^Some AUC values are not available in the publications.

^5^Prediction results of EXIA2 combined with PSI-Blast PSSM.

^6^Prediction results of EXIA2 without evolutionary information.
