# Supplementary material for: EXIA2: Web Server of Accurate and Rapid Protein Catalytic Residue Prediction
Source: Biomed Res Int. 2014 Sep 11;2014:807839. doi: 10.1155/2014/807839 (PMC4177735; doi:10.1155/2014/807839)
Supplement: Supplementary file 1 — Figure S1 Side chain orientation for catalytic and noncatalytic residues of PW79 dataset: Distribution of the angle between residue side chain vector and the vector of residue C alpha atom to the center point of catalytic site (center point of selected residues for noncatalytic residue). Figure S2 Examples of triplet selection and score calculations: Detailed score calculation and triplet selection of an example structure including nine grid points. Figure S3 Comparison of ROC curves of EXIA2 and CRpred: Comparison of ROC curves of EXIA and CRpred on the EF fold dataset. EXIA, with or without including PSSM, performs better than CRpred. Figure S4 Comparison of ROC and RP curves of EXIA2, ResBoost, and ConSurf: Comparison of ROC and RP curves of EXIA, ResBoost, and ConSurf on the P100 dataset. EXIA, with or without including PSSM, performs better than the other two methods. Figure S5 ROC curve of prediction with and without amino acid combination feature on the EX79 dataset: To see the effect of amino acid combination feature on the prediction performance, we compare the AUCROC of prediction using structure feature only and using both structure feature and amino acid combination. The AUCROC is improved from 0.938 to 0.944. Table S1 List of PDB IDs and chain for 55 pairs of structures of the L55 dataset: 55 pairs of enzyme structures selected from the PW79, POOL160, and EF datasets. Each pair contains a structure with bounding ligand and a structure without bounding ligand. Table S2 List of PDB IDs and chain of EX79 dataset: To test the prediction performance on a dataset excluding all structures used for parameter optimization (PW79 dataset), we construct the EX79 dataset by combining all proteins in the POOL160, and EF datasets and excluding all proteins of the PW79 dataset. [file 807839.f1.pdf]

Figure S1 Side chain orientation for catalytic and noncatalytic residues of PW79 dataset

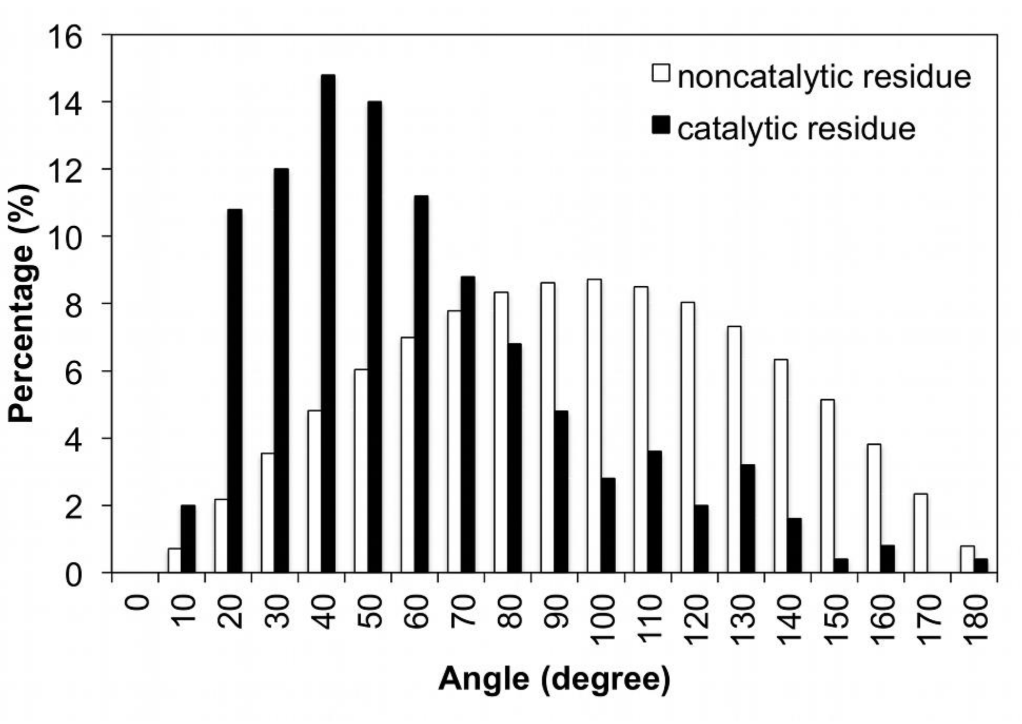

Figure S2 Examples of triplet selection and score calculations

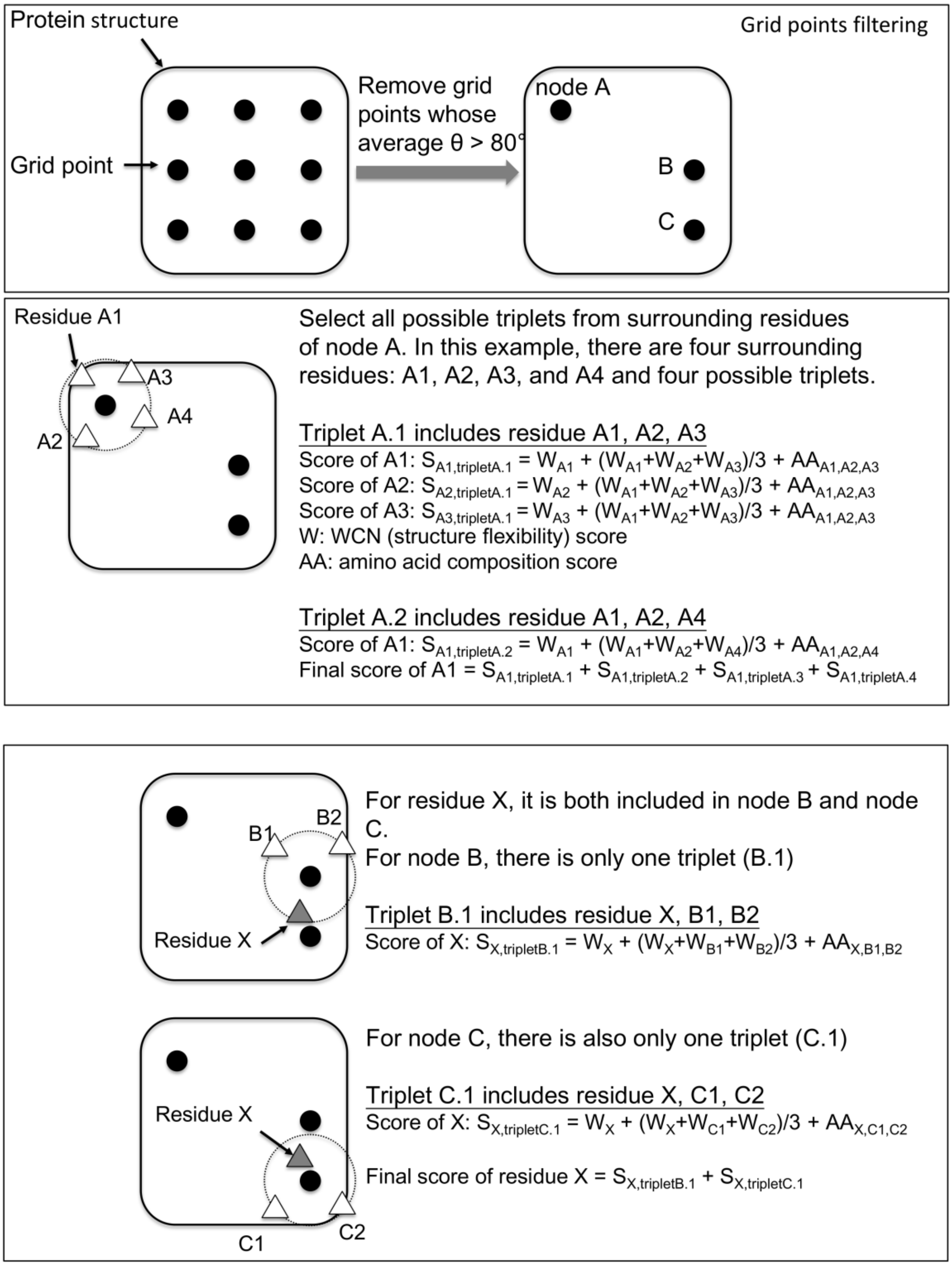

Figure S3 Comparison of ROC curves of EXIA and CRpred

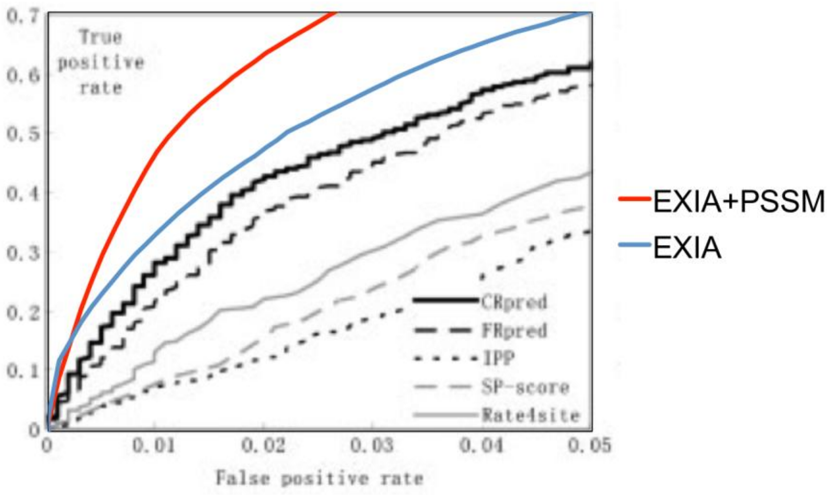

ROC curves of EXIA superimposed with that reported by CRpred.

Figure S4 Comparison of ROC and RP curves of EXIA, ResBoost, and ConSurf

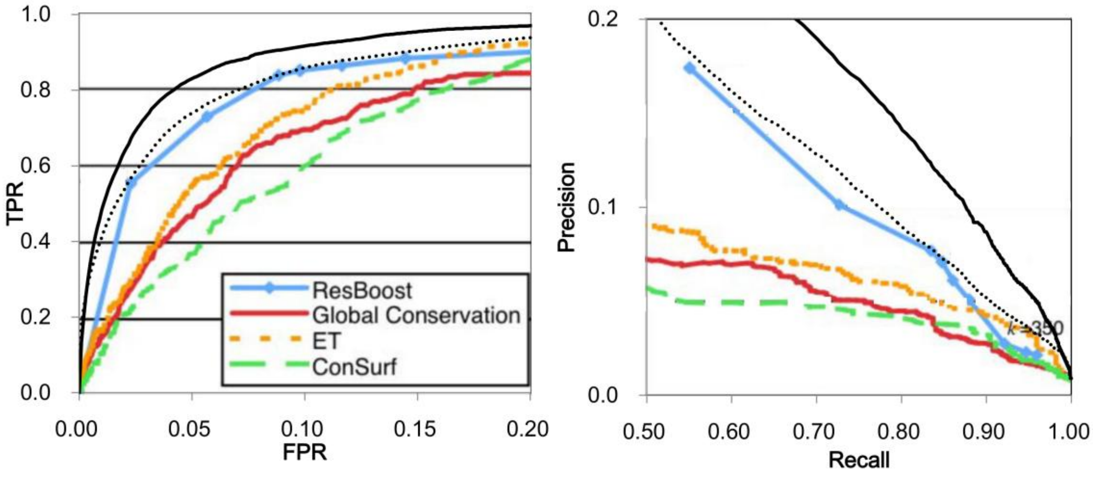

Black solid lines: EXIA+PSSM; black dotted lines: EXIA

Figure S5 ROC curve of prediction with and without amino acid combination feature on the EX79 dataset

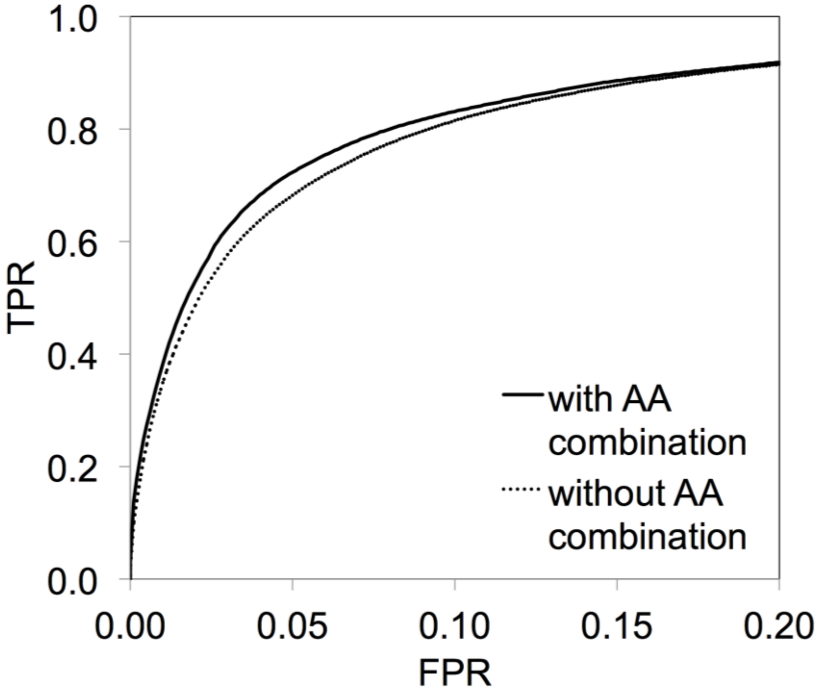

Table S1 List of PDB IDs and chain for 55 pairs of structures of the L55 dataset

**PDB IDs and Chain of L55 Dataset**			
**Unbounded structure**	**Bounded Structure**	**Unbounded structure**	**Bounded Structure**
1a4s_a	1bpw_a	1kra_c	1krb_c
1a4y_b	4aoh_a	1l6p_a	3pfu_a
1a6d_a	1a6e_a	1l7d_a	1f8g_a
1ab4_a	2y3p_a	1lci_a	1ba3_a
1akm_a	1duv_a	1lxa_a	2qiv_x
1ako_a	2voa_a	1m53_a	2pwg_a
1aug_a	3ro0_a	1mla_a	2g2o_a
1auo_a	1aur_a	1pgs_a	1pnf_a
1b65_a	3n2w_a	1pjb_a	1say_a
1b6b_a	1cjw_a	1pma_b	1yar_h
1b73_a	1b74_a	1qfn_a	1grx_a
1bml_a	1l4d_a	1smn_a	1g8t_a
1bo1_a	2ybx_a	1tyf_a	1yg6_a
1brm_a	1gl3_a	1uch_a	1xd3_a
1bwp_a	1wab_a	1uok_a	4aie_a
1c4z_a	ld5f_a	1vie_a	1vif_a
1cd5_a	1fs5_a	1zym_a	3eze_a
1cz1_a	1eqc_a	2alr_a	3cv7_a
1db3_a	1t2a_a	2eng_a	3eng_a
1dco_a	1dch_a	2fok_a	1fok_a
1di1_a	1dgp_a	2plc_a	1aod_a
1esc_a	1esd_a	2pth_a	3vjr_a
1eug_a	3eug_a	1jfl_a	2dx7_a
1f2v_a	1i1h_a	1k30_a	1iuq_a
1fcq_a	1fcv_a	1k32_a	1n6e_a
1fug_a	1rg9_ab	1kas_a	1b3n_a
1g24_a	2a9k_b	1kfu_l	3bow_a
1geq_a	1wdw_a		

Table S2
List of PDB IDs and chains of EX79 dataset

12asA
13pkA
1a05AB
1a0iA
1a41A
1a4yB
1a6dA
1a79A
1a8hA
1a8qA
1aa6A
1ab4A
1adnA
1af7A
1afrA
1ak0A
1akoA
1alkA
1am2A
1apyB
1aq0A
1astA
1augA
1auiA
1aukA
1auoA
1avqA
1ax4A
1b04A
1b5qA
1b5tA
1b65A
1b66B
1b66A
1b6gA
1b73A
1b9hA
1bcrA
1bd0AB
1bd3A
1bf2A
1bfdA
1bg0A

1bg6A
1bhgA
1bjpA
1bmlA
1bmtA
1bo1A
1bobA
1booA
1bouB
1bqcA
1brwA
1bs0A
1bwdA
1bwpA
1c17A
1c3cAB
1c3jA
1c4xA
1c4zA
1cb8A
1cc1LS
1cf2O
1cfrA
1cg2A
1chkA
1chkB
1chmA
1cjwA
1cjyA
1cl1A
1cmxA
1coyA
1cqqA
1cs1AC
1cvrA
1cz1A
1d0sA
1d1qA
1d2tA
1d3gA
1d4aA
1d4cA
1d5rA
1d8cA
1d8hA
1dciA

1dekA
1dgsA
1dhpA
1di1A
1diiA
1dinA
1dkiA
1dl2A
1dl5A
1dliA
1dmuA
1do8A
1dpgA
1dqaAB
1dtwAB
1dw9AF
1dxeA
1e0cA
1e19A
1e1aA
1e2tA
1e5qA
1e7lAB
1eb6A
1ebfA
1ecfA
1ecmAB
1eejA
1eg7A
1ehkAB
1ehyA
1ei5A
1eq2A
1escA
1et0A
1eu1A
1eugA
1eulA
1f2dA
1f2vA
1f6dA
1f75A
1f7lA
1f7uA
1f8mA
1f8rA

1fa0A
1fcqA
1fftF
1fghA
1fgjAB
1fobA
1fohA
1fr2B
1froA
1fugA
1fuiA
1g0dA
1g24A
1g72A
1g8fA
1g8pA
1g99A
1ga8A
1gdhA
1gdoB
1geqA
1getAB
1gogA
1goxA
1gp1A
1gpjA
1gq8A
1gsaA
1gt7A
1gtpA
1gtxA
1h3iA
1h4gA
1h54A
1hdhA
1hfeM
1hfsA
1hr6B
1hrkA
1hv9B
1hzdA
1hzfA
1i19A
1i1iP
1i6pA
1i78A

1i7dA
1i7qAB
1i8dA
1i8tB
1ig8A
1im5A
1inpA
1itxA
1iu4A
1ivhA
1j09A
1j79A
1jchA
1jflA
1jh6A
1jhfA
1jkmA
1jm6A
1jmsA
1jnrAB
1jofA
1jqnA
1k0wA
1k30A
1k32A
1k4tA
1k82A
1kdgA
1kfuL
1kl7A
1knyA
1kwsA
1kyqA
1kzhA
1kzlA
1l0oA
1l1dA
1l1lA
1l6pA
1l7dA
1l9fA
1l9xA
1lamA
1lbuA
1lcbA
1lciA

1ldmA
1lmlA
1ltqA
1lucA
1m53A
1m6kA
1masA
1mekA
1mhlAC
1mhyD
1mkaAB
1mppA
1mpxA
1mt5A
1mucA
1mugA
1nbfA
1nf9A
1ni4AD
1nidA
1nsjA
1nvmA
1nvtA
1nw9B
1nwwA
1nzyA
1o04A
1o8aA
1o98A
1oacA
1oe8A
1ofdA
1ofgA
1otgA
1p3dA
1pbgA
1pgsA
1piiA
1pknA
1pmaB
1pmiA
1ps1A
1psdA
1pvdA
1pyaA
1pyaF

1pyaAEF
1pymA
1qamA
1qazA
1qbaA
1qd1A
1qd6CD
1qdlAB
1qe3A
1qf6A
1qfmA
1qfnA
1qgnA
1qh5A
1qhoA
1qi9A
1qj4A
1qmhA
1qprAB
1qq5A
1qtnA
1qumA
1r51A
1ra2A
1rblA
1reqA
1rhsA
1rptA
1rtuA
1slmA
1smlA
1soxA
1tdjA
1thtA
1tmlA
1tyfA
1uagA
1uaqA
1uasA
1uchA
1uroA
1ushA
1vaoA
1vieA
1vncA
1xyzA

1ytwA
1zioA
1zymA
2abkA
2admA
2ahjAB
2aprA
2ayhA
2bmiA
2dorA
2ebnA
2engA
2fokA
2gsaA
2hdhA
2nacA
2nlrA
2oatA
2pdaA
2pgdA
2phkA
2piaA
2pthA
2sqcA
2thiA
2tpsA
3claA
3csmA
3mddA
3pcaM
4kbpA
5coxA
5enlA
5fitA
7odcA
8tlnE
9papA
